# Role of pelvic packing in the first attention given to hemodynamically unstable pelvic fracture patients: a meta-analysis

**DOI:** 10.1186/s10195-022-00647-6

**Published:** 2022-07-07

**Authors:** Pengyu Li, Fanxiao Liu, Qinghu Li, Dongsheng Zhou, Jinlei Dong, Dawei Wang

**Affiliations:** 1 Department of Interventional Radiology and Vascular Surgery, Peking University First Hospital, 8 Xishiku Street, Beijing, 100034 China; 2 Department of Orthopedic, Shandong Provincial Hospital, Cheeloo College of Medicine, Shandong University, 324 Jingwu Road, Jinan, 250021 Shandong China; 3 Department of Orthopedic, Shandong Provincial Hospital, Affiliated to Shandong First Medical University, 324 Jingwu Road, Jinan, 250021 Shandong China

**Keywords:** Pelvic fracture, Hemodynamic instability, Pelvic packing, Angioembolization, Resuscitative endovascular balloon occlusion of the aorta

## Abstract

**Purpose:**

To evaluate the effectiveness of pelvic packing (PP) in pelvic fracture patients with hemodynamic instability.

**Materials and methods:**

Three databases—PubMed, Embase and the Cochrane Library—were systematically searched to identify studies presenting comparisons between a protocol including PP and a protocol without PP. Mortality, transfusion requirement and length of hospitalization were extracted and pooled for meta-analysis. Relative risk (RR) and standard mean difference (SMD), along with their confidence intervals (CIs), were used as the pooled statistical indices.

**Results:**

Eight studies involving 480 patients were identified as being eligible for meta-analysis. PP usage was associated with significantly reduced overall mortality (RR = 0.61, 95% CI = 0.47–0.79, *p* < 0.01) as well as reduced mortality within 24 h after admission (RR = 0.42, 95% CI = 0.26–0.69, *p* < 0.01) and due to hemorrhage (RR = 0.26, 95% CI = 0.14–0.50, *p* < 0.01). The usage of PP also decreased the need for pre-operative transfusion (SMD = − 0.44, 95% CI = − 0.69 to − 0.18, *p* < 0.01), but had no influence on total transfusion during the first 24 h after admission (SMD = 0.05, 95% CI = − 0.43–0.54, *p* = 0.83) and length of hospitalization (ICU stay and total stay).

**Conclusions:**

This meta-analysis indicates that a treatment protocol including PP could reduce mortality and transfusion requirement before intervention in pelvic fracture patients with hemodynamic instability vs. angiography and embolization. This latter technique could be used as a feasible and complementary technique afterwards.

**Level of evidence:**

3.

## Introduction

Pelvic fractures are often caused by high-energy trauma and have a high mortality, which is always attributable to bleeding [1, 2]. Hemorrhage is the most common cause of death within the first 24 h after injury [3], and the reported mortality rate of patients with hemodynamic instability due to severe pelvic fracture is as high as 40% [4]. Therefore, early recognition and control of the hemorrhage is vital. Multidisciplinary approaches have been used to manage bleeding, including operative management, such as external fixation and pelvic packing (PP), as well as endovascular interventions, e.g., angioembolization and resuscitative endovascular balloon occlusion of the aorta (REBOA). Among these, angioembolization and PP are the most widely used and of the greatest concern.

Angiography and embolization, first discussed in 1972, were reported to have a success rate ranging from 80 to 100% for arterial hemorrhage [4, 5], but showed little effectiveness at controlling venous bleeding [6]. However, arterial bleeding only accounts for 10–15% of cases, and the hemorrhage originates from injured veins or fractured pelvic fragments in more than 80% of patients [7, 8]. Furthermore, the preparation of an angiography suite and a specialized interventional radiologist is time consuming, and delays have been associated with an increase in mortality [9].

Pelvic packing, where the hemorrhage is directly addressed from the retroperitoneal space, was originally described in Germany in 1994 [10, 11]. Contrary to the previous technique, PP can be a quick and effective procedure that is most commonly used for venous bleeding. PP can be performed within 20 min in an emergency room by experienced surgeons [12]. After modification, PP has been widely used in European trauma centers as a salvage procedure for hemodynamically unstable patients with pelvic fractures [11–16]. In Norway, Gaski et al. adopted extraperitoneal PP as part of a formal treatment protocol for severe pelvic injuries in Oslo University Hospital more than two decades ago [16]. Frassini et al. described PP as a life-saving procedure that could be the first step in the multidisciplinary management of pelvic ring disruptions [12]. At the First Italian Consensus Conference, a statement agreed that PP is effective and proposed an algorithm in which PP is performed prior to angiography [17]. Aside from Europe, in the last decade, scholars from China and South Korea have reported improved clinical outcomes since adopting PP in the initial treatment protocol [11, 18–20]. World Society of Emergency Surgery (WSES) guidelines from 2017 recommend that PP should always be considered for patients with pelvic-fracture-related hemodynamic instability, and that maximum effectiveness can be achieved when it is combined with external fixation [21]. However, surgeons in North America seem to be more in favor of angiography and embolization [9]. According to guidelines from both the Eastern Association for the Surgery of Trauma (EAST) and the Western Trauma Association in the United States, angiography remains the mainstay of therapy [22–24].

Institutions from different countries applied PP as part of the treatment algorithm for hemodynamically unstable patients in the twentieth century. The results showed that PP was as effective as angioembolization and that patients may benefit from the change of protocol, as it led to a reduction in mortality and blood transfusion [6, 11, 12, 18–20, 25–27]. A quasi-randomized control study performed in 2014 demonstrated that, compared with angioembolization, pelvic packing had a shorter time to intervention and a shorter surgical time [3]. However, most of these studies were just descriptive and based on small to medium cohorts. Therefore, the efficiency of PP remains controversial due to different outcomes. Although two meta-analyses regarding PP were found in our search of the current literature, one study included only three papers comparing PP with angioembolization [28]. The other was a network meta-analysis with a different aim [29]. We believe a quantitative analysis including a large number of patients would provide more convincing evidence for clinical instruction. The aim of this meta-analysis is to examine the efficacy of early PP in patients with hemodynamic instability due to pelvic fracture. This study hypothesizes that the introduction of PP into the management protocol has a benefit for clinical outcomes in that it lowers mortality and transfusion requirement.

## Materials and methods

This meta-analysis was performed in strict accordance with the Preferred Reporting Items for Systematic Reviews and Meta-analyses (PRISMA) statement/guideline [30].

### Design and search strategy

The search process was performed by two investigators, blindly and independently, using three databases: PubMed, Embase and the Cochrane Library, on March 10, 2020. The complete search terms were: “pelvic packing [All Fields]” AND “pelvic injury [All Fields]” OR “pelvic trauma [All Fields]” OR “pelvic fracture [All Fields].” Additional eligible studies that were missed in the electronic database search were retrieved by screening reference lists. Overall mortality was determined as the primary outcome. Transfusion requirement and length of hospitalization were secondary outcomes.

### Inclusion and exclusion criteria

Included studies had to fulfill all the following criteria: (a) enrolled patients with a pelvic fracture and hemodynamic instability; (b) studies comparing clinical outcomes between patients treated with PP and patients without PP, or studies presenting a comparison of results between a treatment protocol including PP and a protocol without PP; and (c) articles written in English.

Exclusion criteria were: (a) non-original studies (including reviews, meta-analyses, case reports, comments, editorials, letters, correspondence and conference addresses); (b) enrolled patients < 14 years old; and (c) studies with insufficient data for the required indicators to be extracted.

### Surgical technique

The technique was improved by Pohlemann et al. [10] in 1994 to packing of the retroperitoneum, and then modified to ensure direct packing of the pelvic space through a preperitoneal approach [31]. The method is usually performed by making an infra-umbilical midline incision of about 6–8 cm. Skin, subcutaneous tissue and fascia are dissected without violating the peritoneal cavity. Three laparotomy pads are placed below the pelvic brim toward the iliac vessels on each side of the bladder [32]. Revision of PP should be done within 48–72 h [21].

### Data extraction

The following data were extracted from the included studies: the first author’s surname, publication year, country of origin, basic characteristics of the participants (number, age and gender), study design, injury severity score (ISS), and primary and secondary outcomes. All data were independently extracted from eligible publications by two of the authors; in the case of any discrepancies, an experienced orthopedic surgeon was consulted until a consensus was achieved.

### Quality assessment

Quality assessment was performed using the Newcastle–Ottawa scale (NOS) [33], which is usually used to assess the quality of nonrandomized studies in a meta-analysis [34, 35]. The NOS included eight items belonging to three categories: (1) study group selection, (2) comparability of groups, and (3) outcome of interest. Each study was given a score of 1 for each item. Studies with high scores were considered good reports. The assessment was performed by two authors; any disagreements were resolved through discussion between the two assessing authors. A study with a score > 7 was considered to be at low risk of bias [33].

### Statistical analysis

The statistical analysis and production of forest plots were performed by STATA 12.0 (StataCorp, College Station, TX, USA). Heterogeneity was assessed using the* χ*^2^ test and the *I*^2^ test. If *p* > 0.1 and *I*^2^ < 50%, the heterogeneity was considered insignificant and a fixed effects model was used. Otherwise, a random effects model was used. The relative risk (RR) (along with its 95% confidence interval, CI) was pooled for dichotomous variables and the standardized mean difference (SMD) (along with its 95% CI) was pooled for continuous variables. To explore the source of heterogeneity, subgroup analyses were performed according to different factors. Sensitivity analysis was conducted by omitting studies one by one to evaluate the stability of the results. Publication bias was investigated using a funnel plot. A two-tailed *p* value < 0.05 indicated statistical significance.

## Results

### Search results and study inclusion

**Fig. 1:**
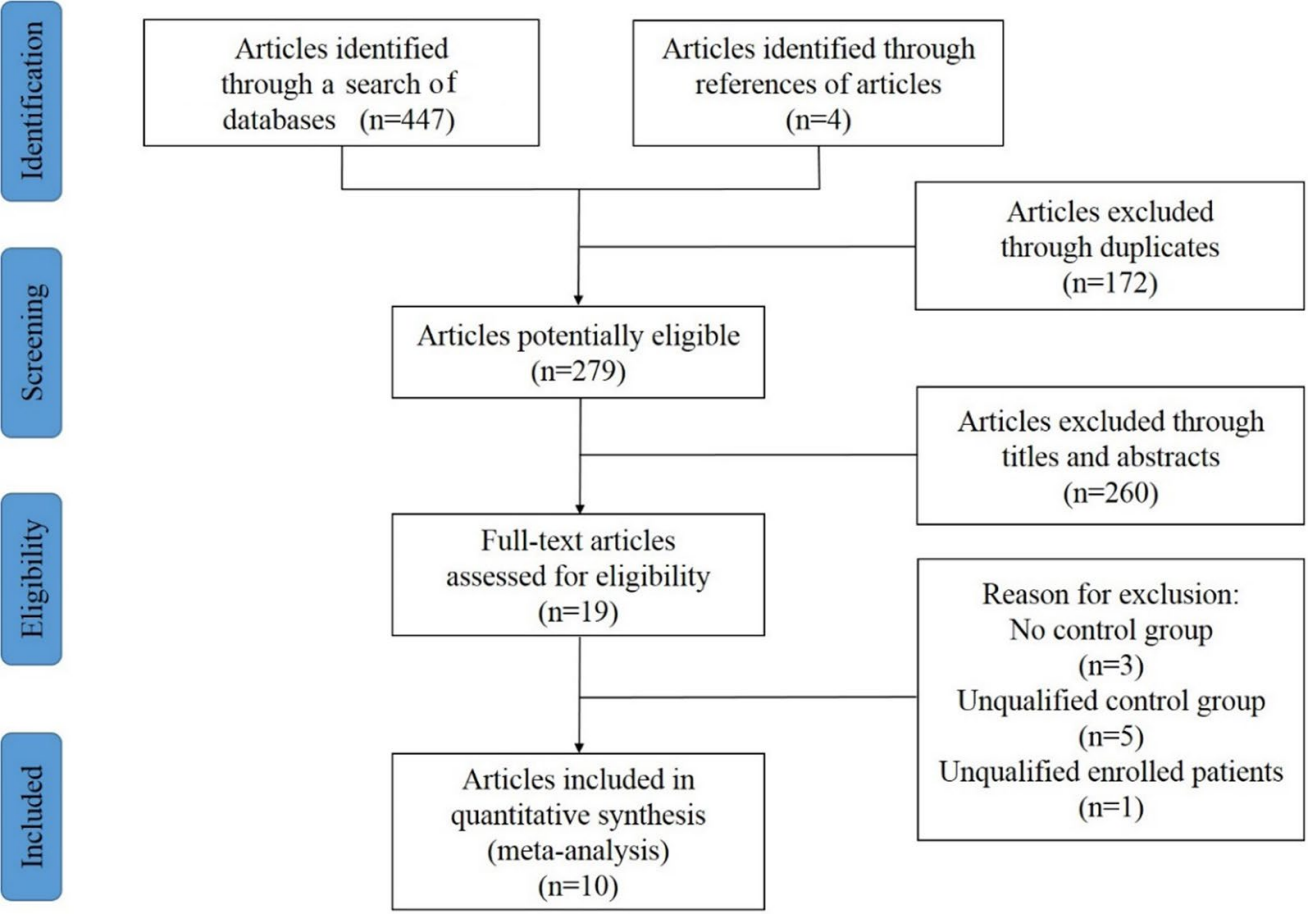
Flow diagram of the selection process for the included studies

A total of 447 records were retrieved from three databases after the initial search. Another four records were identified by reviewing citations in the references. Among the total studies retrieved, 172 were removed because of duplication. Subsequently, 260 articles were excluded after reading the titles and abstracts. Then 19 studies were downloaded and assessed for eligibility by reading the full texts. Eventually, 10 articles [3, 6, 11, 12, 18–20, 26, 27, 36] were considered to qualify for this meta-analysis. The detailed selection process is depicted in Fig. 1.

### Characteristics of the included studies and risk of bias

The basic characteristics of the 10 included studies are summarized in Table 1. All studies were published from 2009 to 2020, including results from Asia [3, 18–20, 26, 27], Europe [11, 12], the United States [6] and Australia [36]. Eight articles [6, 11, 12, 18–20, 26, 27] were retrospective cohort studies and two [3, 36] were prospective studies. Eight studies [11, 12, 18–20, 26, 27, 36] had nine points and two studies [3, 6] had eight points using the NOS score. Of note, studies by Chiara et al. [11] and Frassini et al. [12] and studies by Jang et al. [27] and Shim et al. [19] appeared to contain patients from the same period in the same hospital. We enrolled the latest studies with the largest number of patients [12, 19]. The other two studies were omitted just in case of double counting [11, 27]. The sample sizes of the eight enrolled studies ranged from 24 to 125, and a total of 480 patients were included.

**Table 1:** Main characteristics of the included studies

Author (year)	Country	Study design	No. of patients				Age (years)		ISS		NOS
			Total	PP	Non-PP		PP	Non-PP	PP	Non-PP	
Osborn (2009)	USA	Retrospective	40	20	20		37.9 ± 18.9	39.5 ± 17.4	54.7 ± 12.7	45.9 ± 8.7	8
Tai (2011)	China	Retrospective	24	11	13		51.2 ± 19.0	44.8 ± 24.7	40.0 ± 12.5	42.3 ± 18.8	9
Cheng (2015)	South Korea	Retrospective	125	49	76		45.37 ± 21.02	46.84 ± 21.43	40.10 ± 14.19	45.00 ± 15.71	9
Chiara (2016)	Italy	Retrospective	50	25	25		PSM		PSM		9
Hsu (2016)	Australia	Prospective	24	14	10		49.9 ± 17.5	60.3 ± 23.5	32.0 ± 6.7	23.8 ± 12.7	9
Jang (2016)	South Korea	Retrospective	30	14	16		59.7 ± 15.0	60.9 ± 22.1	38.8 ± 8.3	32.2 ± 4.9	9
Li (2016)	China	Prospective	56	27	29		43 ± 13	40 ± 9	48 ± 6	43 ± 7	8
Lee (2017)	South Korea	Retrospective	79	43	36		53.2 ± 19.8	50.6 ± 19	38.7 ± 12.5	37.2 ± 12.3	9
Shim (2018)	South Korea	Retrospective	58	30	28		62.5 ± 14.4	57.0 ± 22.8	38.4 ± 8.5	38.7 ± 9.2	9
Frassini (2020)	Italy	Retrospective	74	37	37		PSM		PSM		9

*PP* Pelvic packing, *ISS* Injury severity score, *PSM* Propensity Score Matching, *NOS* Newcastle-Ottawa scale

### Mortality

**Fig. 2:**
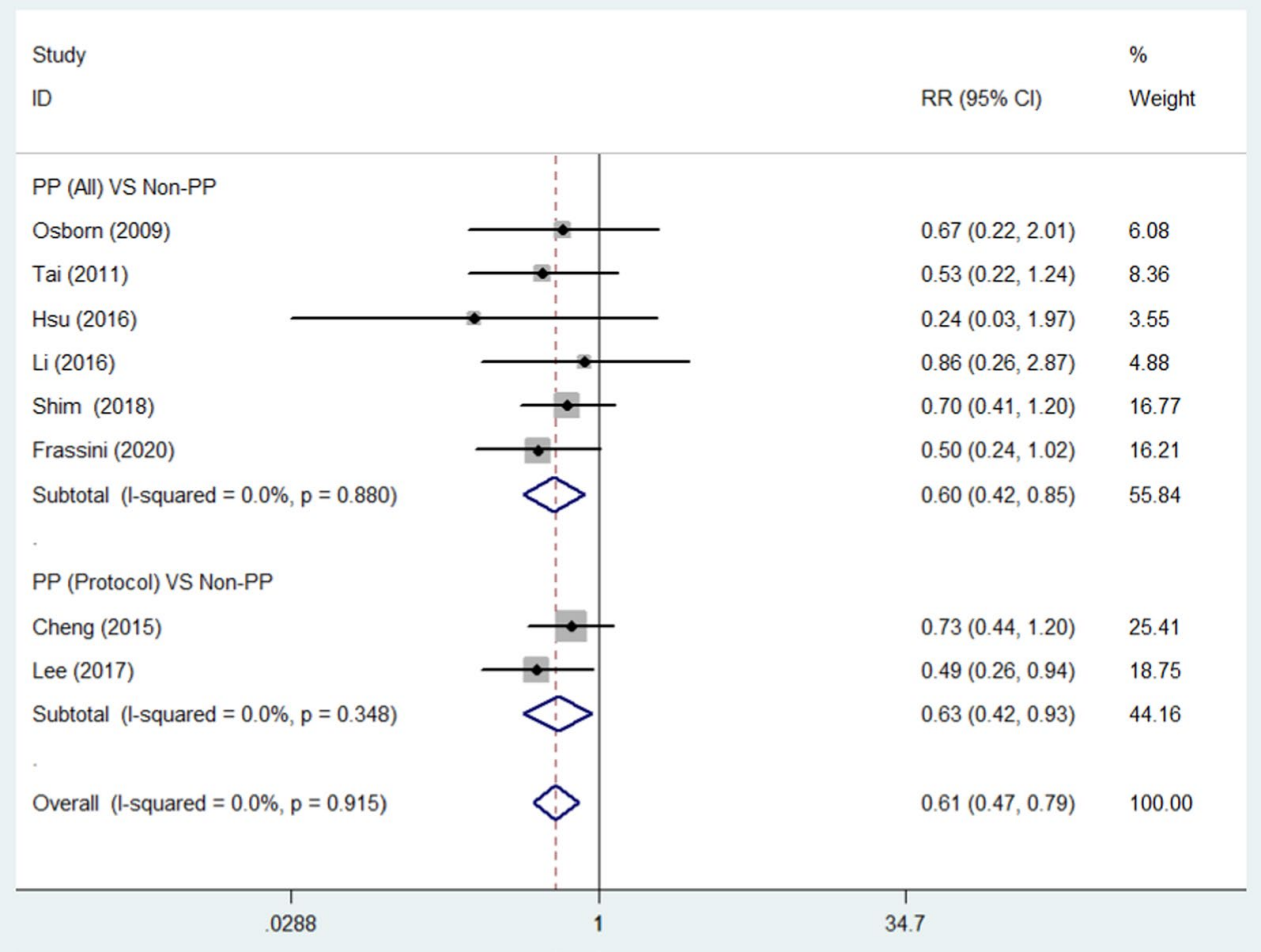
Forest plot involving a comparison of overall mortality. *RR* relative risk, *CI* confidence interval

**Fig. 3:**
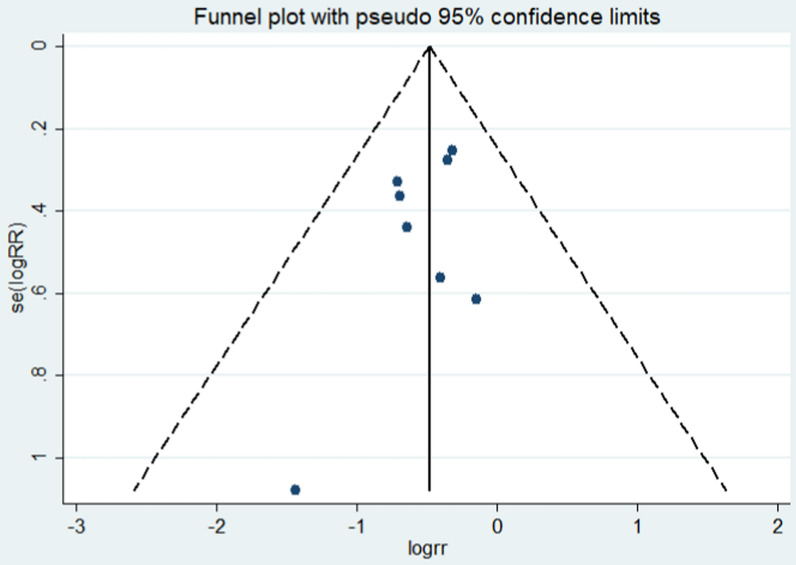
Funnel plot of publication bias. Two studies were omitted just in case of double counting [5, 9]

**Fig. 4:**
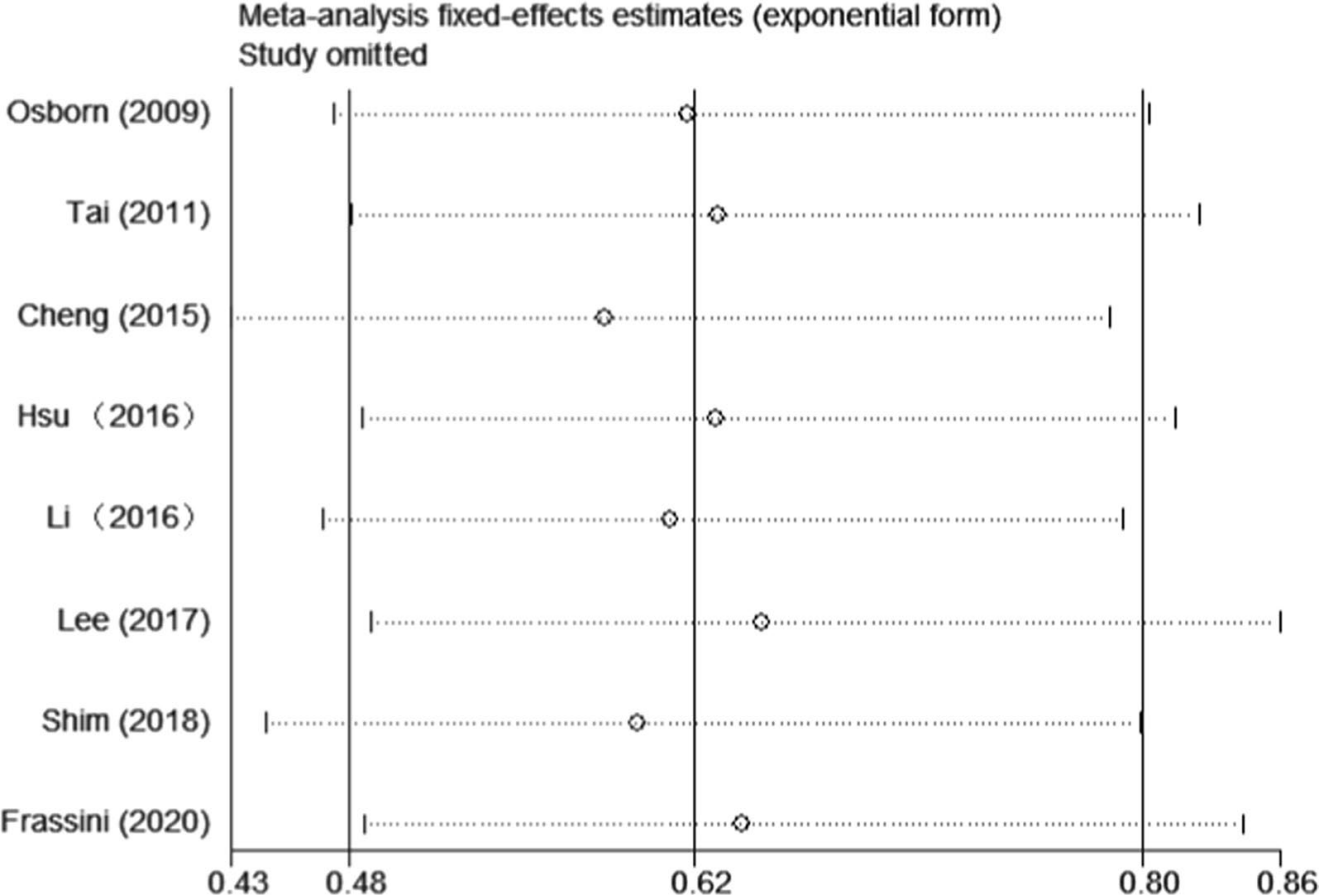
Sensitivity analysis involving overall mortality, performed by omitting studies one by one. Two studies were omitted just in case of double counting [5, 9]

**Fig. 5:**
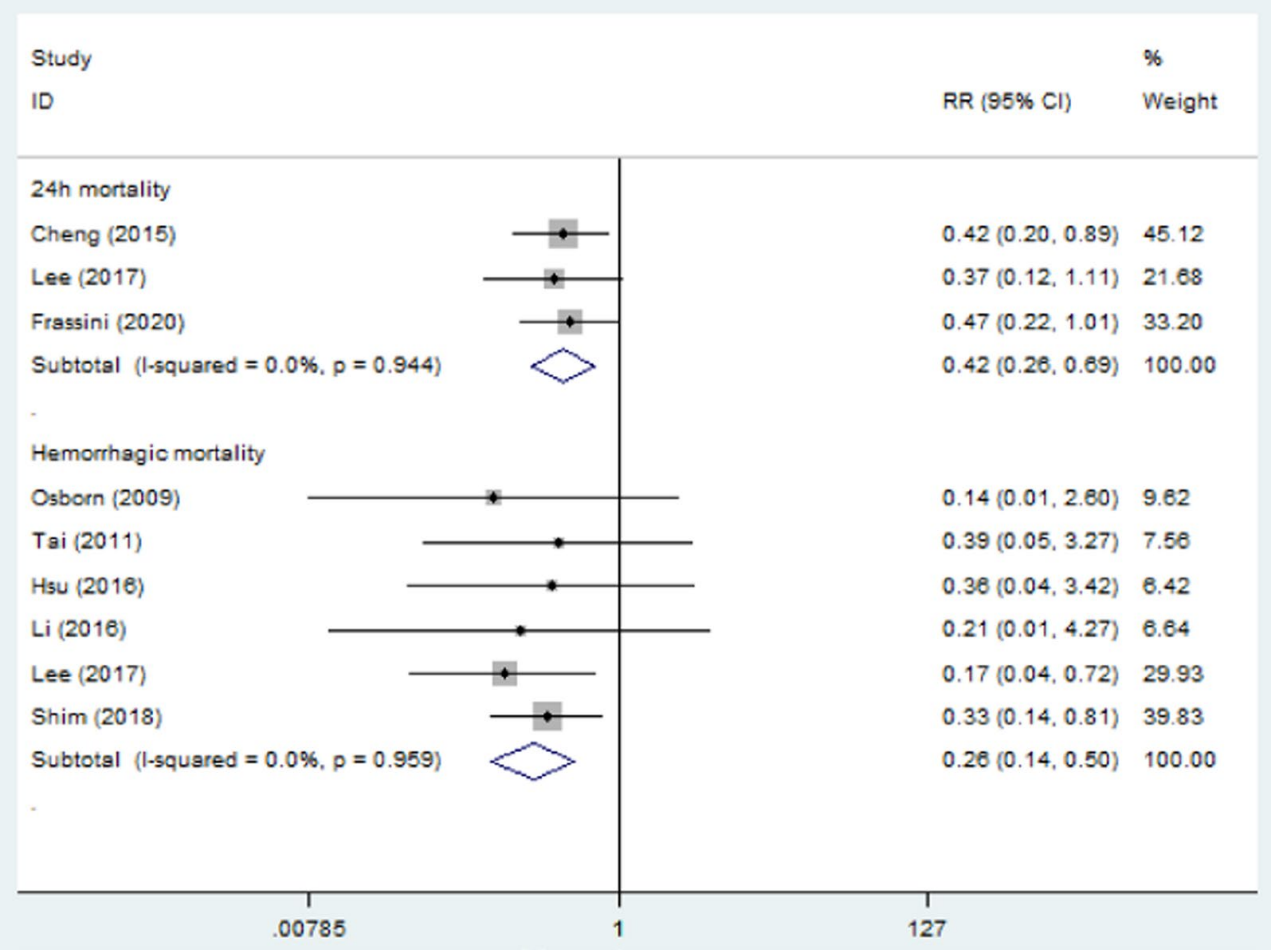
Forest plot involving a comparison of early mortality. *RR* relative risk, *CI* confidence interval

All included studies were evaluated for overall mortality. The mortality was 25.11% (58/231) in the PP group and 41.77% (104/249) in the non-PP group. Overall mortality was significantly lower in the PP group (RR = 0.61, 95% CI = 0.47–0.79, *p* < 0.01) (Fig. 2). The *I*^2^ statistic was 0%, indicating no heterogeneity among the included studies. No significant publication bias was found (Fig. 3). Sensitivity analysis was conducted by omitting studies one by one, which indicated that the results were stable (Fig. 4). Considering that patients from two studies [18, 20] were divided into two groups: those who received a protocol with PP and those who received a protocol without PP, and not all patients in the PP group received pelvic packing, subgroup analyses were performed. The patients treated with PP or just a protocol including PP had reduced mortality rates (RR = 0.60, 95% CI = 0.42–0.85, *p* < 0.01; RR = 0.63, 95% CI = 0.42–0.93, *p* = 0.02). The pooled results from three and six studies showed that PP decreased 24-h mortality (RR = 0.42, 95% CI = 0.26–0.69, *p* < 0.01) and hemorrhagic mortality (RR = 0.26, 95% CI = 0.14–0.50, *p* < 0.01), respectively (Fig. 5). The *I*^2^ statistic was 0% for 24 h and hemorrhagic mortality, indicating no heterogeneity among the included studies.

### Transfusion requirement and length of hospitalization

**Fig. 6:**
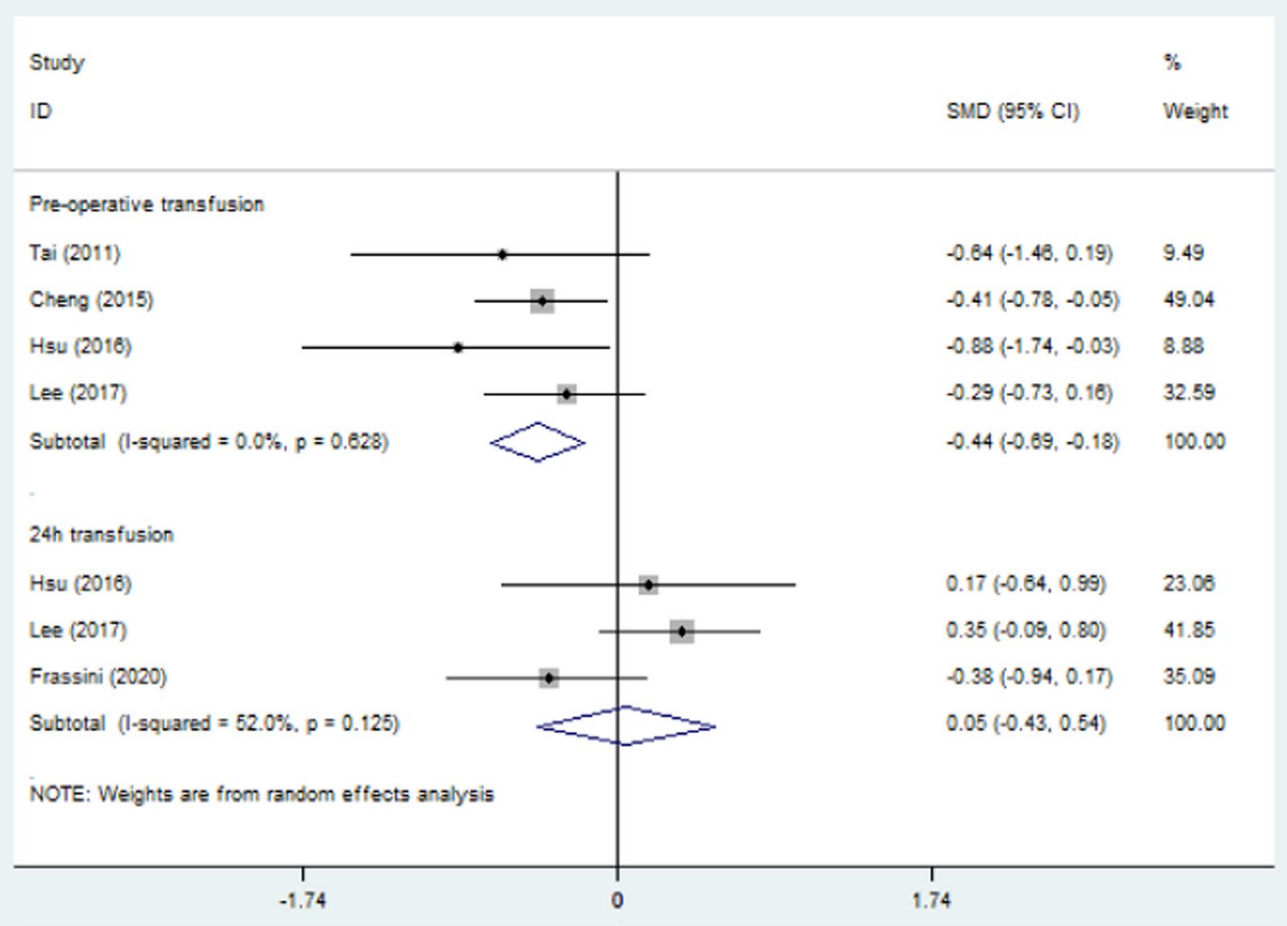
Forest plot involving a comparison of pre-operative transfusion and transfusion during the first 24 h after admission. *SMD* standard mean difference, *CI* confidence interval

**Fig. 7:**
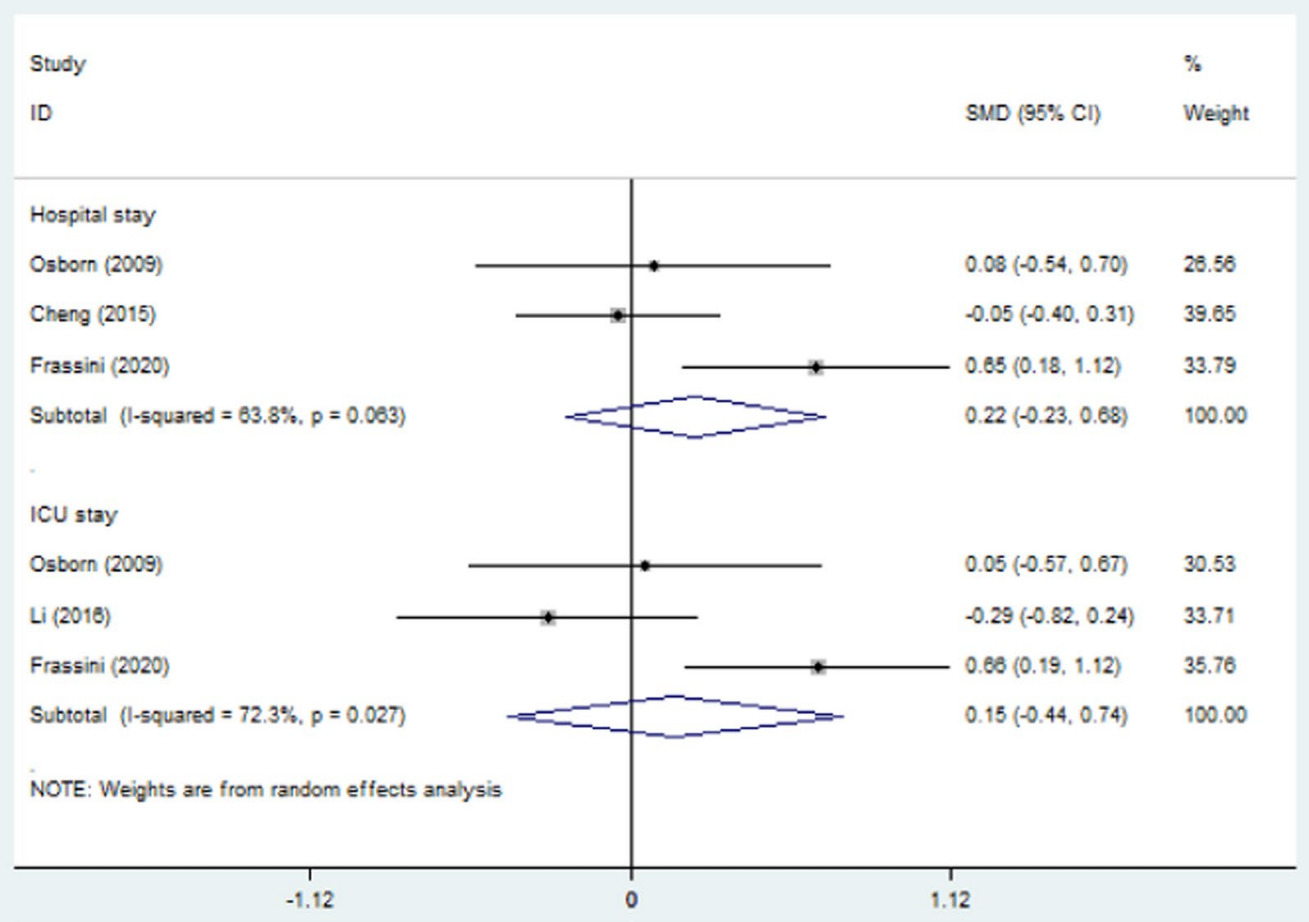
Forest plot involving a comparison of length of hospitalization. *SMD* standard mean difference, *CI* confidence interval

Blood transfusion was measured in packed red blood cell (PRBC) units. The combined results revealed that PP decreased the need for pre-operative transfusion (SMD = − 0.44, 95% CI = − 0.69 to − 0.18, *p* < 0.01; *I*^2^ = 0%) but had no influence on transfusion during the first 24 h after admission (SMD = 0.05, 95% CI = − 0.43–0.54, *p* = 0.83; *I*^2^ = 52.0%) (Fig. 6). Total length of hospital stay and length of stay in ICU were not changed by PP (SMD = 0.22, 95% CI = − 0.23–0.68, *p* = 0.34; SMD = 0.15, 95% CI = − 0.44–0.74, *p* = 0.61, Fig. 7). The *I*^2^ statistic was 63.8% (95% CI 0%–89.6%) for total length of hospital stay and 72.3% (95% CI 0%–89.6%) for ICU stay. The cause of heterogeneity could not be found due to insufficient data, and the random effects model was used.

## Discussion

This meta-analysis revealed that PP use was associated with significantly reduced overall mortality as well as reduced mortality within 24 h after admission and due to hemorrhage. The usage of PP also decreased the need for pre-operative transfusion but had no influence on total transfusion during the first 24 h after admission and length of hospitalization, which indicated that a treatment protocol including PP could reduce mortality and transfusion requirement before intervention in pelvic fracture patients with hemodynamic instability vs. angiography and embolization.

Pelvic packing was originally performed using a trans-abdominal approach after laparotomy, but poor results were initially reported [4, 26]. The disruption of intact peritoneum probably affected the tamponade of the hemorrhage, leading to aggravation of bleeding. A multicenter observational study conducted by the American Association for the Surgery of Trauma (AAST) in 2015, which enrolled patients from 11 Level I trauma centers, demonstrated that the mortality rate was 32% in a series of pelvic fracture patients in shock [37]. Another modern series reported a mortality rate of 27.8% in patients with hemorrhagic instability who were undergoing angiography [9]. Burlew et al. reported a mortality rate of 21% in an 11-year single-center study with a modified protocol that considered PP as the first intervention for pelvic fracture hemorrhage because PP reduced mortality compared with other series favoring angiography and embolization [38]. The updated algorithm from the Western Trauma Association in 2016 also attached more importance to the use of PP [23].

Pelvic packing has the advantages of lowering mortality and reducing time to intervention [18–20, 38], but its results are varied [3, 6, 26]. Its role in the management of pelvic hemorrhage remains controversial, and more studies with a feasible comparison (e.g., angiography) are needed. Only four of the included studies demonstrated that the implementation of PP in the management protocol significantly improved survival [11, 12, 18, 20]. Death within the first 24 h after admission is commonly due to exsanguination, whereas mortality after 24 h is usually from multiple organ failure [10, 39]. Our quantitative synthesis confirmed this finding that the early use of PP is a life-saving procedure in management for patients in hemorrhagic shock. However, it should be noted that the use of PP cannot be simply linked with improved mortality. Gaski’s group reported that the rate of extraperitoneal PP had decreased as the number of hemorrhagic deaths in their institution reduced due to improved hemorrhage control protocol and because pelvic packing was a life-saving procedure employed when initial resuscitation failed and angiography was unavailable [16]. The importance of an effective resuscitation strategy and a multidisciplinary approach cannot be overemphasized.

A delay in hemostatic procedures is associated with increased mortality in patients with pelvic hemorrhage [12]. Every 3 min of delay in the resuscitation room leads to a 1% mortality increase in a hemodynamically unstable patient with blunt abdominal trauma in the first 90 min [40]. Early hemostasis should be done as early as possible. Currently, angiography is still considered the first choice for hemorrhage control in most institutional algorithms [36]. However, the time required for the transportation of patients, the preparation of the angiography suite and the mobilization of trained interventional radiologists is excessive. Osborn et al. reported a mean time to PP of 44 min from ED admission, compared to a mean time of 130 min to the angiography suite [6]. The average time to operative packing reported by Tai et al. was 79 min, compared with 140 min to angiography [26]. Similar results were presented by Jang et al., with the time to intervention in the PP group being 55 min, compared to 194 min in the non-PP group [27]. A previous study indicated that PP had a shorter procedure duration than angiography [3]. Recently, a study from Italy demonstrated that the total hemostatic procedure time was sharply reduced for patients in the PP group, with a mean time of 49 min, compared to 156 min in the non-PP group [12]. As numerous studies have confirmed that PP has the advantages of immediacy and rapidity [3, 6, 12, 20, 26, 27, 36, 38], a quantitative analysis was not performed.

The availability of angiography varies in hospitals. Low-level trauma centers, especially in remote or rural regions, may not be equipped with a certified angiography suite. Meanwhile, interventional radiologists are not present in-house at all times [3], and interventions are easily delayed during nights and weekends [41]. Metcalfe et al. reported that a 24-h formal interventional radiology service was only available at 18% of hospitals in Wales, UK [42]. PP is a fast and easy procedure with a low demand for equipment and short learning curve, and deserves more widespread use. Moreover, the high-energy trauma that causes pelvic fractures often leads to an increased risk of associated injuries. The rapid arrest of the hemorrhage by PP facilitates other emergent operative procedures to stabilize polytrauma patients [18].

Reducing transfusion is a compelling objective, since the need for transfusion is associated with increased length of ICU stay, multiple organ failure and mortality [25]. It was reported that the mortality rate increased by 62% with every one PRBC unit per hour increase in the transfusion rate [43]. In addition, PRBCs may induce adverse inflammatory responses by activating inflammatory genes in circulating leukocytes [44]. With PP included in the protocol, though the total number of transfusions required in the first 24 h after admission did not change, the need for transfusion in the ED was significantly reduced. The reduced time to intervention for PP is critical to the decreased need for pre-operative transfusion. Pelvic fractures are often caused by high-energy trauma, which results in a high probability of associated injuries involving abdominopelvic viscera, major vessels, limbs and even the head. These associated injuries may lead to a need for extra transfusion. Osborn et al. reported that packing significantly decreased blood transfusion over the 24 h post-intervention period, whereas angiography demonstrated no such change [6]. Burlew’s group also reported a significant reduction in the transfusion requirement after PP [25, 38]. However, previous studies demonstrated that post-intervention transfusion was similar in patients treated with PP or with angiography [3, 26]. A quantitative analysis was not performed on account of inadequate data. Further studies are needed to assess the role of PP in blood transfusion requirement.

Although the use of PP improves survival, it cannot completely replace angiography and embolization. During the initial resuscitation in pelvic trauma, it is difficult to accurately ascertain the source of bleeding [26]. Therefore, the optimal procedure may be hard to determine in a short time. Since the primary source of pelvic bleeding is injured veins or fractured bone and angiography is time consuming, PP could be considered the first-line treatment for pelvic fracture patients with unstable hemodynamics. If patients have sustained hemodynamic instability after PP, arterial bleeding should be suspected, and angiography is necessary. A complementary association of pelvic packing and endovascular procedures seems to be the best clinical practice based on guidelines from the WSES and Western Trauma Association [12]. Suzuki et al. proposed PP as the primary procedure for patients with unstable hemodynamics, whereas angiography could be the first choice for stabilized patients [45]. Totterman et el. suggested that PP could be supplemented with angiography once sufficient hemodynamic stability had been attained [14]. It should be pointed out that, based on current evidence, it is unclear whether secondary angiography should be performed on all patients or just on those who still have a manifestation of continuous bleeding after PP.

In recent years, REBOA has been proposed as an alternative for temporary bleeding control in hemodynamically unstable trauma patients [21]. REBOA has the advantage of rapidly and effectively controlling an arterial hemorrhage while preserving cerebral and myocardial perfusion [23]. WSES guidelines and the Western Trauma Association suggest that REBOA may act as an effective adjunct in the management of hemodynamically unstable pelvic ring injuries. However, the occlusion time is associated with ischemia–reperfusion injury and amputation. Currently, REBOA is mainly considered as a bridge from emergent hemostasis to a secondary procedure [12].

Several limitations of this study are now listed. First, only two of the included studies were prospective studies, and no randomized controlled trial was included. However, a randomized study was not reasonable for ethical and practical reasons. Second, data for accessing transfusion requirement were limited. Third, propensity score matching (PSM) analysis was used to adjust for differences in the baseline characteristics between the two groups in the two studies [11, 12], and we only enrolled patients after PSM. The neglected data might affect the strength of the conclusions.

## Conclusions

This meta-analysis indicates that a treatment protocol including PP significantly reduces mortality and transfusion requirement before intervention in pelvic fracture patients with hemodynamic instability. Pelvic packing is recommended as a feasible method for patients with traumatic pelvic hemorrhage that is complemented by angiography and embolization.

## Abbreviations

PP: Pelvic packing
RR: Relative risk
SMD: Standard mean difference
CIs: Confidence intervals
REBOA: Resuscitative endovascular balloon occlusion of the aorta
PRISMA: Preferred Reporting Items for Systematic Reviews and Meta-analyses
ISS: Injury severity score
NOS: Newcastle–Ottawa scale
PRBC: Packed red blood cells
WSES: World Society of Emergency Surgery
EAST: Eastern Association for the Surgery of Trauma
AAST: American Association for the Surgery of Trauma
PSM: Propensity score matching
